# Ophthalmic Complications of Periorbital and Facial Aesthetic Procedures: A Literature Review

**DOI:** 10.7759/cureus.41246

**Published:** 2023-07-01

**Authors:** Maryam M Alharbi, Mohammed S Bin Dlaim, Jawza M Alqahtani, Najd S Alkhudhairy, Shahad M Almasoudi, Nouf T Alajmi

**Affiliations:** 1 Department of Clinical Sciences, College of Medicine, Princess Nourah bint Abdulrahman University, Riyadh, SAU; 2 Department of Ophthalmology, King Abdullah bin Abdulaziz University Hospital, Princess Nourah bint Abdulrahman University, Riyadh, SAU

**Keywords:** hyaluronic acid filler, ophthalmic complications, facial laser, facial fillers, facial injections, rhinoplasty, surgeries, procedures, ocular

## Abstract

The emergence and popularity of cosmetic facial procedures may lead to significant ophthalmic complications such as ocular motility dysfunction and visual disability. Here, we present a scoping review to identify common ophthalmic complications in some facial plastic surgeries and cosmetic injections, and to develop clinical approaches for prophylaxis and management in terms of direct attention and awareness of non-ophthalmologists toward such scenarios and appropriate intervention. This review was conducted according to the Preferred Reporting Items for Systematic Reviews and Meta-Analyses (PRISMA) guidelines. The following keywords were used to search PubMed, Scopus, Web of Science, and Google Scholar: “facial laser”, “facial fillers”, “facial injections”, “hyaluronic acid”, “local facial injections of botulinum toxin”, “rhinoplasty”, “blepharoplasty blindness”, “ophthalmoplegia”, “diplopia”, “ptosis”, “ophthalmic artery occlusion”, “posterior ciliary artery occlusion”, and “ocular ischemic syndrome”. A total of 37 articles published between 1989 and 2021 were included, of which 21 were case reports. The most common ophthalmic complication was vision loss (0.0008%). The risk of ophthalmic complications including ocular pain, sudden unilateral or bilateral vision loss, flashes of light, ptosis, and ophthalmoplegia increase with injection in common anatomical regions like the glabella, nose, and supraorbital and nasolabial folds. The incidence of adverse events ranges from 5% to 18% in rhinoplasty. The most common complications after blepharoplasty were dry eye syndrome and diplopia, caused by eyelid ptosis. Eyelid, cornea, lens, and retina injuries are ophthalmic complications that occur after facial laser treatment. Ophthalmic complications after non-ophthalmic and cosmetic procedures are becoming increasingly common. The cumulative reported cases of ophthalmic complications after hyaluronic acid filler injection from 2016 to 2020 showed different types of adverse events, with the most common being decreased visual acuity, unilateral vision loss, and ptosis, with varying outcomes of each complication ranging from partial resolution to complete recovery. These complications must be recognized early, and prompt treatment must be established.

## Introduction and background

Devastating ophthalmic complications, including impairment or loss of visual acuity, can result in catastrophic outcomes after cosmetic injections and facial procedures. The incidence of visual loss in general following non-ophthalmic procedures is estimated to range from 0.056% to 1.3%, depending on the type of surgery [1]; however, permanent visual loss can occur, accounting for 0.0008% [2]. Several factors increase the risk of ophthalmic complications after non-ophthalmic surgeries, including age and sex, chronic diseases such as long duration in the prone position, excessive blood loss, hypotension, anemia, hypoxia, excessive fluid replacement, use of vasoconstricting agents, elevated venous pressure, head positioning, and a patient-specific vascular susceptibility that may be anatomic or physiologic. However, the risk factors for any given patient or procedure may vary and are likely multifactorial [3]. If, when an ophthalmologist is consulted for a patient with perioperative visual loss, an obvious ocular cause is not apparent, urgent neuroimaging should be obtained to rule out intracranial pathology. Anterior and posterior ischemic optic neuropathy (ION) should be considered and careful documentation is essential. Furthermore, hematocrit and blood pressure should be maintained within the patient’s tolerances and the duration of surgery should be kept short. Etiologies of postoperative ophthalmic complications include external ocular injury or corneal abrasion, cortical blindness, central retinal artery occlusion, and ION [4].

Postoperative ocular complications of non-ophthalmic surgeries affecting the orbit include corneal abrasion, enophthalmos, emphysema, and most commonly orbital hemorrhage. Detection and proper management will prevent visual loss due to ischemia, complications extending to the extraocular muscle causing ocular motility dysfunction, unilteral or bilateral irreversible blindness if it reaches deep into the optic nerve, and other non-serious complications that might affect the lacrimal drainage system [5]. The purpose of this review is to identify the possible causes from previously published studies and case reports of common ophthalmic complications of some facial plastic surgeries and cosmetic injections, in addition to developing approaches for prophylaxis and management.

## Review

Methods

**Figure 1:**
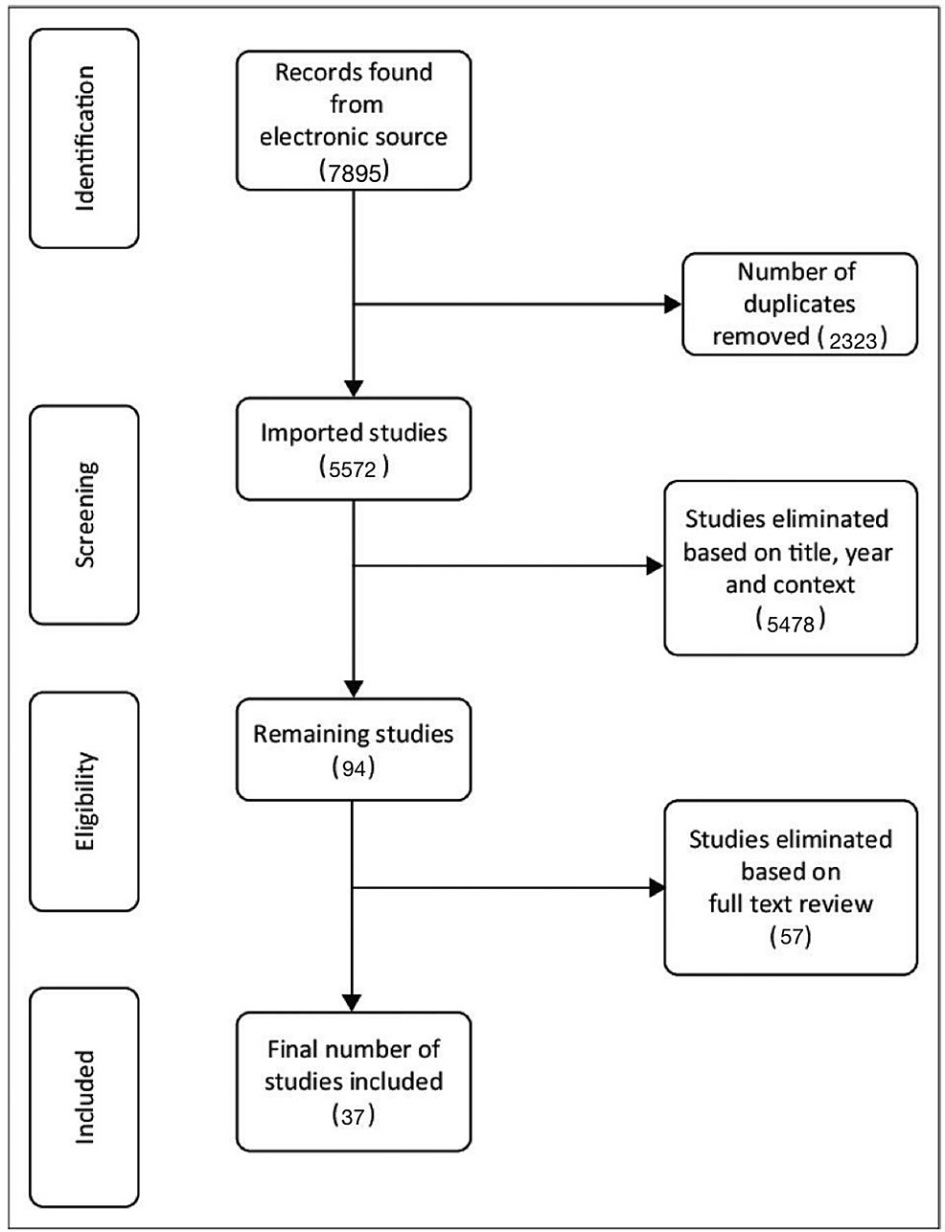
PRISMA flow diagram for included studies PRISMA: Preferred Reporting Items for Systematic Reviews and Meta-Analyses

This scoping review was conducted according to the Preferred Reporting Items for Systematic Reviews and Meta-Analyses (PRISMA) guidelines. We searched the literature for studies published between 1985 and 2021 to establish reported cases of ocular complications after hyaluronic acid (HA) filler injections and to determine the possible causes, management, and prevention of such complications. PubMed, Scopus, Web of Science, and Google Scholar were searched using the following terms and keywords: “facial laser”, “facial fillers”, “facial injections”, “hyaluronic acid”, “local facial injections of botulinum toxin”, “rhinoplasty”, “blepharoplasty blindness”, “ophthalmoplegia”, “diplopia”, “ptosis”, “ophthalmic artery occlusion”, “posterior ciliary artery occlusion”, and “ocular ischemic syndrome” (Figure 1).

Results and discussion

HA Filler Injections

There are numerous case reports but few research studies in the literature regarding ophthalmic complications after non-ocular surgeries or procedures. With the common use of facial injection in plastic and aesthetic surgery, there is a possibility of rare, severe, and devastating ophthalmic complications. Lately, there has been an increase in the use of soft tissue fillers, which can be justified by modern beauty standards and are in global demand because of their influence on an individual's self-confidence, psychological state, and personal and professional growth [6]. Injectable fillers have highly predictable and reproducible outcomes and are safe and fast. However, they are associated with some serious side effects. According to a study conducted in 2015, permanent ( Autologous fat) and semi-permanent (HA) fillers together caused visual complications with a rate of 71.4% [7]. And according to a review study, most of the side effects can be caused by incorrect injection techniques or by the characteristics of the filler [8]. Autologous fat injections are associated with a higher diffuse occlusion rate, poor visual prognosis, and a greater incidence of combined cerebral infarction than HA injections [9]. Thus, it is imperative that patients are mindful of any potential ocular symptoms, common complications after HA, ophthalmoplegia, and complete recovery from ptosis. Nausea and/or vomiting are central nervous system (CNS) complications, including stroke-like features, such as unilateral weakness or evidence of brain infarction on imaging [10].

**Figure 2:**
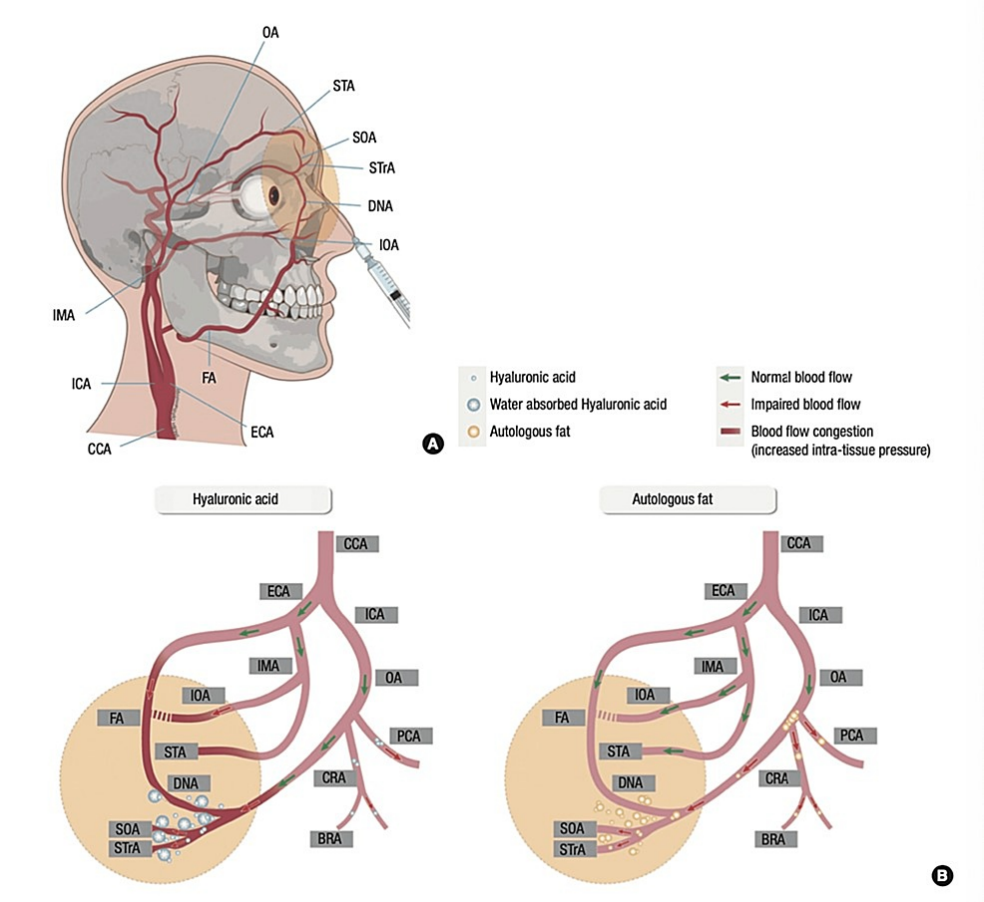
Illustration of vascular anatomy comparing the possible obstruction mechanism between hyaluronic acid and autologous fat (A) This figure illustrates all branches of the external carotid artery that supply the face and might get obstructed if hyaluronic acid or autologous fat gets injected directly into these branches. (B) This figure illustrates all branches of the external carotid artery with normal blood flow or impaired blood flow after injecting hyaluronic acid or autologous fat into those vessels with the increase of intra-tissues pressure in case of obstruction with hyaluronic acid. Image credit: Kim et al., 2015 [19] with permission.

**Figure 3:**
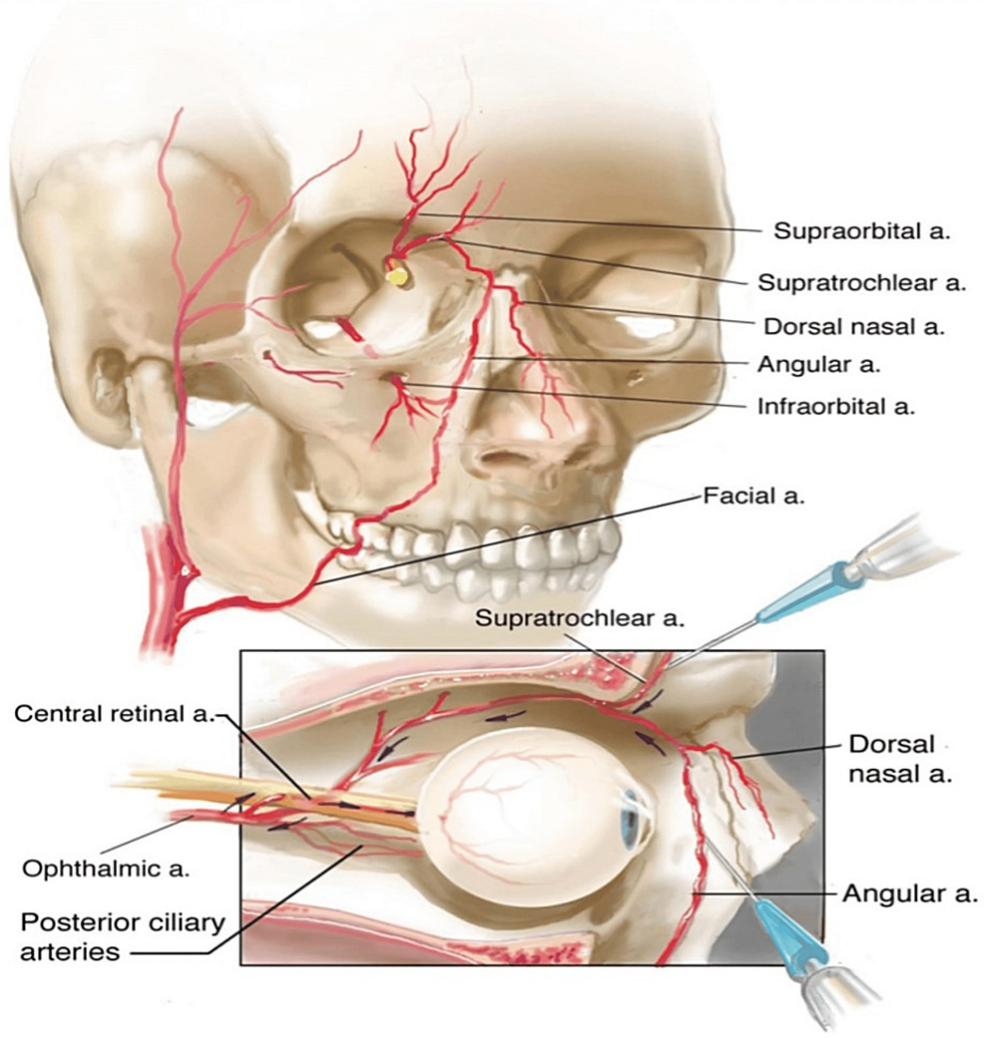
An illustration of the vascular anatomy of the face with selected facial vessels highlighted. Needle positions show potential means of inducing blindness due to filler injection. In this diagram, the filler is injected directly into the supratrochlear artery, in addition to the angular artery, where it anastomoses with the supratrochlear artery, from which it can travel retrogradely (shown by arrows) into the ophthalmic artery and its branches, leading to blockage of the blood supplying the retina and causing visual adverse events. Image credit: Beleznay et al., 2019 [10] with permission.

The proposed underlying mechanism assumes intravascular injection and retrograde embolization of the filler, as the injected bolus may overcome arterial pressure and move against the direction of blood flow. When the injector releases the plunger, the filler travels with blood flow and enters the ophthalmic artery and its branches. With higher injection pressures, filler particles may be pushed further retrograde and enter the brain circulation, which in turn results in cerebral infarction (Figure 2A) [11]. Detailed knowledge of the vascular anatomy (Figure 3), risk factors, and safe injection techniques are required of injectors. In addition, they must be able to recognize the warning signs and symptoms of vascular compromise, get the patient's informed consent, and start a treatment plan immediately if complications arise (Figure 2). The ophthalmic artery begins behind the eye and branches into the supraorbital, supratrochlear, and dorsal nasal arteries. When high-risk sites are injected, such as the glabella, nose, and forehead, there is a risk of intra-arterial filler injection. However, due to the complex vascular anatomy of the face, essentially any location of the facial region may be at risk of ocular complications [7]. There were seven case reports published from 2016-20 involving these types of complications: all patients had injectable HA filler for cosmetic causes, injected at the glabella and nose [12], eyelid [13], forehead [14,15], nose [16,17], and glabella, dorsum, and the tip of the nose [18]. Initial symptoms varied between cases but generally included eyelid swelling, periorbital or orbital pain, ptosis, diplopia, unilateral or bilateral vision loss, and CNS symptoms such as headache, dizziness, upper limb weakness, and ocular complications (Table 1). After initiating treatment, two of the seven patients showed no improvement in ocular complications [14,18].

**Table 1:** Studies reporting complications after hyaluronic acid filler injection.

S. No.	Study	Type of study	Type of filler	Site of injection	Initial symptoms	Complications	Management	Outcome
1	Kapoor et al., 2020 [12]	Case report	HA	Glabella and nose	Periorbital pain and headache	Decreased visual acuity, pain, ptosis, ecchymosis, unilateral upper limb weakness	Hyaluronidase	Complete improvement within half day
2	Loh et al., 2018 [13]	Case report	HA	Eyelid	Eyelid swelling	Vertical diplopia on the left side and downgaze, lower eyelid swelling	Hyaluronidase	Complete improvement within half day
3	Kim et al., 2020 [14]	Case report	HA	Forehead	Right upper limb weakness	Vision loss in the left eye, ocular pain, headache, limb weakness	Thrombolysis, corticosteroids, oxygen therapy, the formula for optic nerve nourishment	No improvement
4	Shi et al., 2018 [15]	Case report	HA	Forehead	Vision loss and orbital pain	Vision loss, ptosis, black and purple discoloration of the nasal dorsum	Hyaluronidase, 2 h of daily hyperbaric oxygen therapy, oral aspirin, oral acetazolamide, and intravenous dexamethasone	Partial improvement
5	Fang et al., 2018 [16]	Case report	HA	Nose	Diplopia, ptosis, and dizziness	Decreased visual acuity, ptosis, dilated pupil, decreased extraocular muscle movement, ecchymosis/discoloration/swelling of the forehead and nasal dorsum	Aspirin, nicegorline, systemic steroid pulse, hyaluronidase (intradermal), topical antibacterials	Partial improvement
6	Hu et al., 2016 [17]	Case report	HA	Nose	Diplopia and orbital pain in the right eye	Decreased visual acuity, ptosis and ophthalmoplegia in the right eye, skin necrosis	Hyaluronidase, hyperbaric oxygen, acetazolamide, ginkgo biloba, cobamamide, dexamethasone tract of ginkgo biloba, cobamamide, and dexamethasone	Partial improvement
7	Sharudin et al., 2019 [18]	Case report	HA	NA	Vision loss	Vision loss in the right eye	Hyaluronidase, hyperbaric oxygen, acetazolamide, ginkgo biloba, cobamamide, dexamethasone tract of ginkgo biloba, cobamamide, and dexamethasone	No improvement

 *Autologous Fat Injections*

Table 2 shows there are four case reports involving autologous fat injections into the glabellar area [20], nasal region and nasolabial fold [21], left side of his nose, each nasolabial fold, and the upper and lower lips [22], supraorbital area [9], and forehead [9]. Immediately after injection, symptoms included sudden unilateral or bilateral vision loss [9-20], ocular pain [9], light flashes [9], headache, ptosis, ophthalmoplegia [20], headache, and painless visual loss [22]. In contrast to the cases reported for HA, only one patient showed full improvement.

**Table 2:** Studies reporting complications after autologous fat injections.

S.No.	Study	Type of study	Type of filler	Site of injection	Initial symptoms	Complications	Management	Outcome
8	Szantyr et al., 2017 [9]	Case report	Autologous fat	Right supraorbital and right forehead area	Ocular pain, flashes of light	Complete visual loss of the right eye	Alprostadil 40 ug, intravenous dexamethasone	No improvement
9	Dreizen and Framm, 1989 [20]	Case report	Autologous fat	Glabella	Ptosis, ophthalmoplegia	Vision loss in the left eye, ocular pain,	Corticosteroids	Complete improvement
10	Park et al., 2008 [21]	Case report	Autologous fat	Right nasolabial fold	Visual loss in right eye	Decreased visual acuity, ptosis, and whitish patchy lesions with macular edema	High-dose steroid therapy (methylprednisolone 1 g)	Partial improvement after 3 months
11	Danesh-Meyer et al., 2001 [22]	Case report	Autologous fat	Left side of his nose, each nasolabial fold, and the upper and lower lips	Eye pain with visual loss	Permeant visual loss	Not reported	No improvement

Treatment and management: There is no standard management protocol for ocular or ophthalmic complications after filler injection. However, the movement of extraocular muscles, signs of ptosis, visual acuity by the chart, and swinging flashlight test for pupillary reaction should be evaluated, and management should be delivered urgently in patients with compromised vascular supply to prevent retinal ischemia. For HA fillers, hyaluronidase should be injected at the same sites as the filler along the path of the anastomosing arteries, and the area of the supraorbital or supratrochlear notch could be considered to cannulate the arteries and push the hyaluronidase retrogradely [23]. Other treatments can be conducted either in the clinic, such as topical timolol, ocular massage, or oral aspirin [24], or by an ophthalmologist or an appropriate specialist, including intravenous acetazolamide, mannitol, anterior chamber paracentesis [24], prostaglandin [25], sublingual glyceryl trinitrate, hyperbaric oxygen, or direct intravascular or intravenous injection of hyaluronidase with urokinase [26]. Heparin, systemic steroids, and antibiotics have also been considered [24].

Additionally, in a retrospective study of ophthalmic and retinal artery occlusions after cosmetic facial filler injections, a cerebral angiographic imaging technique was used to illustrate the features of these adverse events according to the type of injected filler. Interestingly, all patients included in this study had diffuse occlusion of the ophthalmic artery and its branches. Furthermore, all cases were similar in having diminished choroidal and retinal perfusion on fluorescein angiography. However, on selective ophthalmic angiography, all patients injected with autologous fat showed large filling defects primarily in the proximal portion of the ophthalmic artery. In contrast, patients injected with HA had ophthalmic artery obstruction at a distal site compared to those who received an autologous fat injection. Moreover, 69% of patients with HA injections developed skin necrosis in comparison to 23% of patients with autologous fat injections. The most favorable explanation for this phenomenon is that direct vascular obstruction caused by HA filler injections results in decreased angiographic overflow in the distal branches of the internal maxillary and facial arteries. We concluded that these angiographic characteristics were attributed to differences in the size of the injected material, as HA has smaller and more uniform particles than large aggregated autologous fat particles. This causes a more proximal obstruction owing to its larger size rather than the distal obstruction found in HA particles. Moreover, HA increases blood flow to the injected area owing to its hydrophilic and volume-expansion properties [19]; Clearly, complications of HA can be superficial or skin deep causing skin edema or necrosis, or deep, affecting ocular and brain blood supply and causing embolic infarction, as reported about in a case of unilateral blindness due to iatrogenic ophthalmic artery occlusion accompanied by bilateral brain infarction after cosmetic facial filler injection at glabella area [27].

**Figure 4:**
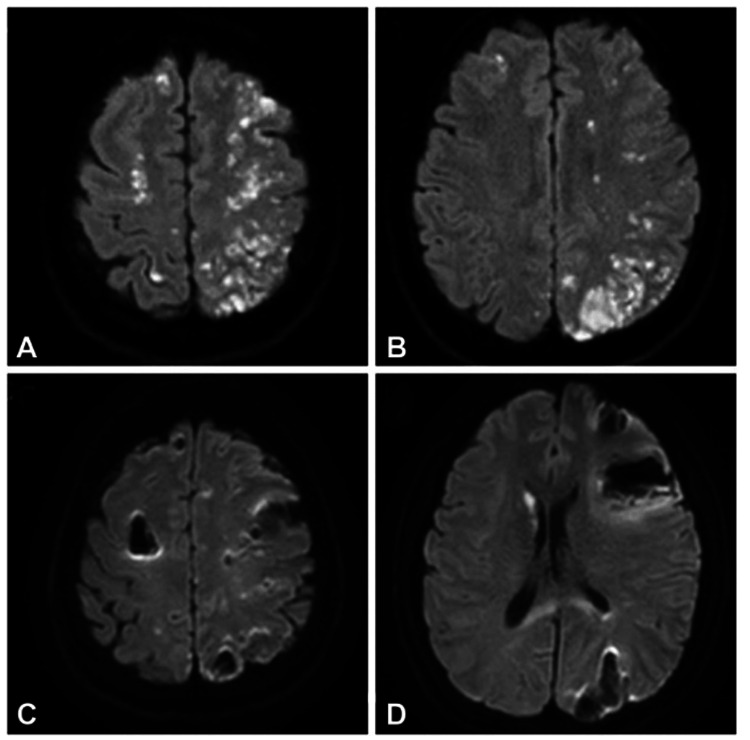
MRI brain image showing acute embolic infarction after facial hyaluronic acid filler injection into the glabella area (A, B) Brain diffusion MRI on day 1 after facial filler injection showing numerous high signal-intensity lesions in both cerebral hemispheres, involving the frontal, parietal, and occipital lobes. These were acute embolic infarcts caused by filler-related emboli. (C, D) In follow-up diffusion MRI on day 7, extensive hemorrhagic transformation that could be categorized as parenchymal hematoma was noted in the previous infarcted foci, especially in both frontal and left parieto-occipital lobes. Image credit: Lee et al., 2021 [27] with permission.

Rhinoplasty

Ophthalmic complications after non-ophthalmic surgery are rare. However, the overall incidence of adverse events after rhinoplasty ranges from 5% to 18% [28]. Orbital complications of rhinoplasty can be vascular, traumatic infectious. Swelling may impair lacrimal drainage and can be demonstrated radiographically for three months. Laceration of the lacrimal sac can cause acute purulent dacryocystitis and should be treated with intubation of the lacrimal system. An enophthalmos, developing months after aesthetic rhinoplasty, is a rare complication. Permanent negative pressure by occlusion of the maxillary ostium may cause a “silent-sinus syndrome” and the contraction of the cavity of the maxillary sinus can result in a displacement of the orbital content. Streptococci can cause a necrotizing periorbital or orbital infection. It is essential to distinguish between “normal” postoperative swelling and periorbital infection. In cases of pain, fever, and pathologic blood parameters, systemic application of penicillin may prevent orbital cellulitis and lid necrosis. Blindness following rhinoplasty is mostly caused by occlusion of the central retinal artery. Also, its embolization as a result of intranasal injection of local anesthetics has to be considered [29,28]. Furthermore, there are numerous well-established risk factors associated with the occurrence of ophthalmic complications after rhinoplasty, such as injection of local anesthetic into the artery while doing mucosa injection during surgery can lead to occlusion of the vessels of the optic nerve. There are a few cases in which visual loss followed the injection of local anesthetic into the nasal mucosa during septorhinoplasty, whereas lateral osteotomies cause most of the sudden orbital-periorbital complications.

**Figure 5:**
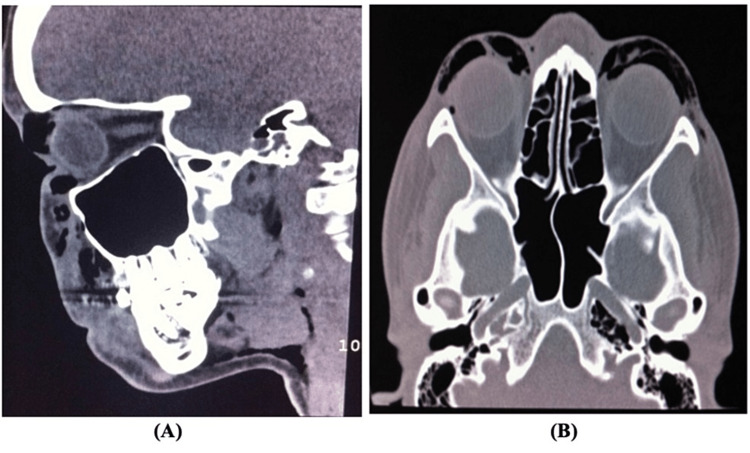
CT brain image showing per-orbital emphysema without crepitation after closed-structured rhinoplasty. (A) CT (sagittal) showing emphysema in the per-orbital area. (B) CT (axial) showing emphysema in the per-orbital area. Image credit: Charles-de-Sa et al., 2014 [29] with permission.

The shearing or tearing of the tissue involving the angular vessels can cause orbital hemorrhage. Because of the confined space of the orbit, significant orbital hemorrhage can increase intraocular pressure (IOP). Regular IOP is necessary for normal vision. The aqueous formation and drainage affect the IOP. The IOP elevation can cause cataracts, iris atrophy, corneal edema, and optic nerve atrophy. Sensation of pressure in the eyes, epiphora, blurred vision, headache, nausea, or vomiting are the symptoms of increased IOP [28]. However, there are other rare complications related to the orbit and periorbital such as periorbital subcutaneous emphysema, its treatment is conservative. The increased pressure can be associated with high-pressure mask ventilation or with gagging or gasping during recovery. It is also hypothesized that air leakage to the orbital region can lead to visual loss via compressive optic neuropathy. Although the incidence of such an event is rare, a case report was published (summarized in Table 3) of a 35-year-old woman with no significant medical history who developed subcutaneous periorbital emphysema after undergoing closed-structured rhinoplasty immediately after extubation, which was interfered with by the patient’s agitation and Valsalva maneuvers [29]. This was followed by bilateral periorbital swelling without crepitation. The diagnosis was confirmed by computed tomography of the head in sagittal and axial slices showing emphysema in the periorbital area (Figure 5). Periorbital swelling was limited and resolved one week after surgery [29]. On the contrary, a study conducted to determine the associated effects of septorhinoplasty on the retina and intraocular pressure (IOP) found that there was no significant effect on the retina or IOP [28]. These complications include periorbital ecchymosis and orbital hemorrhage due to lateral osteotomy, which could lead to increased IOP because of the confined space of the orbit, eventually leading to vascular compromise, resulting in either optic nerve or retinal ischemia. Lastly, adverse events in the periorbital area can be prevented by cautious anesthetic injections and correct osteotomy [28].

**Table 3:** Complications after rhinoplasty with references to studies.

Complication	Cause	Avoidance	Management
Periorbital edema	Inflammation and hemorrhage in soft tissue	Maintaining intraoperative mean arterial blood pressure (50-60mmHg) Preoperative intravenous dexamethasone [30]	Steroids Nasal decongestant
Periorbital ecchymosis	Lateral osteotomy	Maintaining intraoperative mean arterial blood pressure (50-60mmHg) Preoperative intravenous dexamethasone [30]	Conservative management as it resolves spontaneously within 2–4 weeks [29]

Blepharoplasty

Blepharoplasty is one of the most common cosmetic procedures, and its cosmetic and functional aspects are the main reasons for its performance. In most cases, the complications of blepharoplasty are minor, transient, and rarely major or permanent. However, patients must be informed about possible risks before surgery. Complications of blepharoplasty include dry eye syndrome (DES), lower eyelid malposition, diplopia, volume depletion, hematoma, ptosis, and chemosis as mentioned in Table 4. According to a study at Indiana University, the incidence of DES is 26.5% [31]. The symptoms of dryness usually worsen at night because of eyelid shortening caused by incomplete occlusion of the eyes [32]. Predisposing factors for DES include intraoperative canthopexy, postoperative temporary lagophthalmos, concurrent upper and lower blepharoplasty, and transcutaneous approaches that violate the orbicularis oculi muscle. Preoperative history of DES, eyelid laxity, scleral show, or hormone therapy use are also risk factors [31]. DES can be prevented by preoperative screening and the detection of high-risk patients. Dry eyes can often be treated with artificial tears, such as methylcellulose and massage, and they usually resolve spontaneously. Secondly, blepharoplasty may cause mal-positioning of the lower eyelids. According to a study performed in Brazil, six patients out of 200 had malpositioning of the lower eyelids (3%), which varied from a retraction of the lower eyelids in 83% to eversion of the lower eyelids associated with ectropion in 17% of patients. The most common causes of lower eyelid malpositioning include excessive skin resection (anterior lamella) and inadvertent scarring of the orbital septum (middle lamella). Patients who are prone to this complication include elderly adults, those with laxity and deep eye sockets, and those with bulging eyes and negative vectors (anterior portion of the eye bulb anterior to the lower eyelid and the malar promontory) [33]. This complication can be prevented using the trans-conjunctival approach by carefully removing excess skin from the lower eyelid, suturing the orbital septum to avoid inadvertent scarring, and anchoring the lateral cantus, either with canthopexy or canthoplasty [34]. It can be corrected using a suspension of the myocutaneous flap, a free skin graft, or even an upper eyelid flap, and a posterior lamellar expander may also be needed in some cases [33].

**Table 4:** Studies reporting complications after blepharoplasty.

Incidence	Type of study	Publication year	Complications	Management
26.5% [31]	Retrospective	2013	Dry eye syndrome	Treated with artificial tears such as methylcellulose and massage; usually resolves spontaneously
26.3% [31]	Retrospective	2013	Chemosis	Spontaneously heals within 3–4 weeks, vasoconstrictor and steroid eye drops may be used to speed up the healing process
3% [33]	Retrospective	2011	Lower eyelid mal-positioning	Can be corrected by using a suspension of the myocutaneous flap, a free skin graft or even an upper eyelid flap; posterior lamella expander may also be needed in some cases
23.2% [35]	Prospective cohort	2020	Diplopia	Conservative management. In refractory cases, strabismus surgery is required.

However, the members of the American Society of Ophthalmic Plastics and Reconstructive Surgery, who responded to a survey about persistent diplopia after lower blepharoplasty, reported that they had at least one case of persistent diplopia after lower blepharoplasty with a total rate of 23.2% with all surgeons patients’ together [35]. Most cases were vertical, but there is a case report of a 61-year-old female undergoing bilateral upper blepharoplasty who developed horizontal diplopia 24 hours later [36]. There are several causes of transient diplopia, including an edematous reaction, hematoma, trauma with muscle paralysis, and muscular toxicity from local anesthesia. Permanent diplopia is usually caused by direct trauma to muscles or nerves. Lower eyelid blepharoplasty can cause torsional diplopia if the medial and central fat pads are injured. The patient was conservatively managed until no further improvements were observed [36]. Strabismus surgery is required in refractory cases. Fortunately, most cases of diplopia are temporary and resolved without consequence [37].

Finally, chemosis (defined as infiltrative edema of the conjunctiva) can occur as a complication of blepharoplasty, with a prevalence of 26.3% [31]. Liquid collects around the cornea owing to inflammation, lymphatic drainage obstruction, and insufficient eye closure, resulting in a bulging and reddish border. Conjunctival damage occurs more frequently during canthoplasty because of the intense handling of the conjunctiva. However, it spontaneously heals within three or four weeks, and vasoconstrictors and steroid eye drops may be used to speed up the healing process [33]. Table 4 shows four studies reporting complications after blepharoplasty.

Botox

The main complications secondary to botulinum toxin (Botox) injections on the upper face are: ptosis of the eyelid or eye-brow, eyebrow asymmetry, diplopia, lakeophthalmos, palpebral ectropion, and prominence of the palpebral bags. To avoid such complications, it is necessary to have knowledge of the anatomy of this region and adequate and individualized planning based on the existing patterns of the frontalis muscle, glabella, and crow’s feet. Eyelid ptosis can occur in the first two weeks after injecting the procerus muscle, where Botox spreads through the orbital septum, causing a risk of paralysis to the levator palpebrae superioris muscle. The risk of eyelid ptosis can be lowered with experienced injectors and by using concentrated solutions of Botox, which decreases the migratory potential. Diplopia is another possible complication after Botox injection, in which Botox infiltrates the adjacent extraocular muscles. The risk factors for diplopia are the same as those for ptosis. Strabismus and unilateral deviations are severe forms of extraocular muscular dysfunction. Finally, recent studies have demonstrated that a decrease in tear production can occur after injecting Botox into the lateral canthal fold [38].

Facial Laser

Laser depilation has become extremely common as aesthetic lasers have become less expensive and more widely available. Four types of lasers are used for aesthetic purposes: alexandrite (755 nm), diode (800-810 nm), neodymium-doped yttrium aluminum garnet (Nd:YAG;1064 nm), and intense pulsed light lasers (590-1200 nm) [39]. In general, laser hair removal appears to have few adverse side effects, ranging from minor to severe, with few permanent consequences. A study that was done by the Canadian Community Health Survey (CCHS) which collected data from 19,765 Canadians revealed that cosmetic laser treatments (such as removing hair or tattoos) were responsible for most injuries or discomfort (39.1%) [40]. Ocular injuries after laser depilation include eyelid injury, corneal injury, lens injury (cataract), and retinal injury (hypo- or hyperpigmentation, vitreous/ pre-retinal/sub-retinal hemorrhage, retinal pigment epithelium changes, chorioretinal scar, full-thickness macular hole, and choroidal neovascularization). Eyelid injuries included lid retraction and lagophthalmos. In a report of two cases by Miedziak et al., one was of a 73-year-old woman with bilateral redness, tearing, photophobia, and ocular pain after undergoing CO_2_ laser skin resurfacing four weeks prior to presentation [41]. An examination revealed bilateral lower lid retraction, mild ptosis, and lagophthalmos. The patient was treated with aggressive lubrication, nighttime lid tape, and bilateral punctal plugs. The second patient was a 68-year-old woman who reported a decrease in visual acuity in her right eye and had undergone a full facelift 20 years previously, bilateral upper and lower blepharoplasty 10 years previously, and a combined facelift and CO_2_ laser total face skin resurfacing one year previously. She demonstrated bilateral lower lid retraction, mild ptosis, and mild lagophthalmos, which were present bilaterally. She was treated with artificial tears every two hours and bilateral lower lid punctal plugs. In addition, both cases had interpalpebral punctate epitheliopathy on the cornea [41]. This may be because of laser resurfacing, which can cause tightening of the skin around the eyes, pulling the lids down, and inadequate lid closure. Corneal injuries include bullous keratopathy, intrastromal bleeding, corneal burns with scarring, corneal ulcers, exposure keratopathy, and epithelial punctate keratopathy [42]. This may be due to direct laser contact with the cornea as well as exposure to keratopathy, which may be caused by neurological or mechanical disorders that result in widening of the palpebral fissure and/or inadequate lid closure [41,42]. Evaporation of tear film is directly related to the amount and quality of tears produced, as well as the exposed surface area of the eye. Additionally, collagen fiber shrinkage can cause a reduction of up to 25% in the surface area of treated skin, which may lead to secondary lower lid retraction [41]. Management with topical lubricants, punctal occlusion, and taping or patching at night can help [41,42]. 

In addition, patients who underwent alexandrite laser treatment of the eyelid developed pupillary distortion, iris atrophy, iritis, and anterior uveitis. One case report described a 29-year-old Caucasian female complaining of photophobia, an irregular oval pupil, and blurred vision in her left eye after undergoing cosmetic alexandrite laser therapy in the left upper eyelid area without protective eye shields [43]. The patient was diagnosed with acute anterior uveitis and treated with topical corticosteroids. However, within two weeks of follow-up, she developed a marked anterior chamber inflammatory reaction and keratoprecipitation, increased IOP, pigment dispersion over the iris surface, and deteriorated best-corrected visual acuity (BCVA). TRUSOPT® (Merck & Co., Inc., Rahway, New Jersey, United States), an IOP-lowering agent, and intensive steroid treatment with triamcinolone acetonide (40 mg), loteprednol etabonate, and sub-tenon injection was administered. The IOP returned to normal, and the ocular inflammation subsided gradually. Melanin-containing structures absorb energy, leading to depigmentation or photoepilation, and cosmetic lasers may potentially damage the eyes because of the high levels of melanin in the retina and iris. Generally, as the laser wavelength increases, the laser penetration becomes deeper [43].

Furthermore, there is a risk when using lasers to treat the periocular area: they may damage the iris, retinal pigment epithelium (RPE), and choroid because they contain chromophores that can absorb laser energy. In addition, Bell’s phenomenon and thinner eyelid skin expose pigmented eye structures to the laser’s range of action [39]. There is a case report of a 39-year-old man who complained of metamorphopsia and vision loss in his left eye after undergoing a facial laser hair removal session the week before using a 1064-nm Nd:YAG laser. Retinography of the left eye revealed perifoveal hyperpigmentation. During laser hair removal, the laser beam reaches the subfoveal RPE, leading to RPE detachment and fluid exudation into the sub-retinal space. Treatment with one to five doses of intravitreal bevacizumab or ranibizumab has shown excellent outcomes [39]. Recently, a 49-year-old female patient developed macular burns following alexandrite laser epilation. After three months of observation, the visual acuity and fundus findings did not change despite the administration of topical steroids, cyclopentolate, non-steroidal anti-inflammatory eye drops, and systemic non-steroidal anti-inflammatory drugs (in pill form). Permanent scotomas and depression require antidepressant medication and psychotherapy [44]. None of the patients reported wearing goggles to protect their eyes. Hence, owing to the increasing use of lasers in cosmetic settings and the possible associated injuries, adequate and effective education of aestheticians and patient-wear protective goggles are very important.

## Conclusions

Complications following facial and periocular cosmetic and non-ophthalmic procedures are common and increasing, and numerous cases have been reported regarding this concern. We aimed to set a proper management plan and appropriate protocols to be strictly followed in order to prevent and hinder catastrophic adverse outcomes of non-ophthalmic, facial, and periorbital cosmetic procedures. Although permanent visual loss is rare, numerous studies and reported cases have shown that it can occur following many different types of procedures. Therefore, it is crucial to establish a standard management protocol for each procedure, a thorough evaluation of the extraocular muscles, and other domains such as signs of ptosis, visual acuity testing, and pupillary reaction testing. Assessing the signs and symptoms of vascular supply compromise is extremely important to prevent retinal ischemia.
